# Initial pain score during viscous lidocaine instillation and clinical characteristics as predictors of overall pain in fusion prostate biopsy

**DOI:** 10.14440/jbm.0230

**Published:** 2025-10-27

**Authors:** Gabriela M. Diaz, Lindsey T. Webb, Soum D. Lokeshwar, Ankur U. Choksi, Michael S. Leapman, Preston C. Sprenkle

**Affiliations:** 1 Department of Urology, Yale School of Medicine, Yale University, New Haven, Connecticut 06510, United States of America; 2 Department of Urology, University of Connecticut Health, Farmington, Connecticut 06030, United States of America; 3 Department of Urology, Yale New Haven Hospital, New Haven, Connecticut 06510, United States of America; 4 Department of Urology, Veterans Affairs Connecticut Healthcare System, West Haven, Connecticut 06516, United States of America

**Keywords:** Prostate biopsy, Pain, Analgesia, Pain management, Discomfort, Pain predictors, Prostate Cancer

## Abstract

**Background:**

Prostate biopsy, while essential, often causes discomfort that can affect patient experience and adherence to follow-up procedures.

**Objective:**

This study aimed to identify factors associated with pain during fusion prostate biopsy to optimize the experience of prostate cancer diagnosis and monitoring. The primary goal was to assess the relationship between pain during viscous lidocaine (lido) instillation and periprostatic nerve block with the overall pain experienced by patients undergoing prostate biopsy.

**Methods:**

We queried our database for patients who underwent transrectal magnetic resonance imaging-ultrasound fusion prostate biopsy from March 2020 to July 2023 and had complete pain scores (1–10) recorded during lido instillation, periprostatic nerve block, biopsy, and overall.

**Results:**

A total of 779 patients were included. The mean pain scores during lido instillation, periprostatic block, biopsy, and overall were 0.11, 2.8, 3.5, and 3.6, respectively. Multivariable analysis revealed that patients with a pain score during lido instillation of >2 (odds ratio [OR] = 10.28; *p*=0.027) patients with periprostatic block of >2 (OR = 7.49; *p*<0.001), black patients (OR = 2.838; *p*<0.001), and patients on active surveillance (OR = 1.648; *p*=0.003) were more likely to experience the upper quartile (UQ) of overall pain. Men with abnormal digital rectal examination (DRE) findings were less likely to develop the UQ of overall pain than men with normal DRE findings (OR: 0.586; *p*=0.004). This finding suggests that digital rectal examination during the initial clinic visit can help identify patients who may benefit from sedation during prostate biopsy, potentially improving patient comfort and procedural experience.

**Conclusion:**

This finding suggests that digital rectal examination during the initial clinic visit can help identify patients who may benefit from sedation during prostate biopsy, potentially improving patient comfort and procedural experience.

## 1. Introduction

Prostate needle biopsy has been the gold standard for the diagnosis and risk stratification of prostate cancer. The advent of multiparametric magnetic resonance imaging (MRI) has enabled high-resolution prostate imaging, improving the detection of clinically significant prostate cancer previously missed on systematic biopsies, while reducing the detection of indolent cancers.^1^,^2^ These advances in early diagnosis enhance staging sensitivity, thereby providing clinicians with greater support when recommending appropriate management options.

With the rising use of active surveillance (AS) and ablative therapy, a growing number of patients may undergo repeated biopsies. In recent years, the transperineal biopsy approach has gained preference over other approaches due to improved patient safety, lower risk of rectal bleeding, reduced sepsis rates, and better access to the anterior prostate, resulting in higher cancer detection rates.^3^ However, given the associated pain and discomfort, this approach may increase resource use, as patients often require sedation or operating room settings, and therefore, it remains not superior to the transrectal route, as demonstrated in the Prostate Biopsy Efficacy and Complication trial.^4^,^5^

The pain associated with a prostate biopsy is subject to numerous factors. While a direct correlation exists between an increase in the number of punctures and total pain, psychological variables and clinical factors have also been investigated as potential contributors to higher pain, but the literature remains inconsistent.^6^ Although various methods, ranging from pharmacological interventions to non-invasive and cost-effective strategies, have been used to enhance patient comfort, strategies to improve the overall prostate biopsy experience are still underexplored.

Certain factors positively influence patient compliance with AS, including reduced prostate-specific antigen (PSA) levels, lower tumor stage, older age, limited education, and strong social support.^7^ Nevertheless, pain and discomfort during subsequent biopsies may cause some patients to forgo necessary monitoring and reduce adherence to AS. Although non-invasive follow-up protocols demonstrated superiority to radical treatment, their efficacy depends on accurate comprehension of disease progression, which may be compromised if patients are unable to follow up.^8^ Furthermore, pain and discomfort experienced during a prostate biopsy may preclude participation in surveillance or awake biopsies and contribute to AS biopsy non-compliance.^9^,^10^ To identify patients who may benefit from biopsy under sedation, this study aimed to determine prognostic factors associated with overall pain during the performance of fusion prostate biopsy.

## 2. Materials and methods

### 2.1. Study design and patient selection

Our Institutional Review Board approved, prospectively collected database included patients who underwent transrectal MRI–ultrasound fusion prostate biopsy using either ExactVu (Exact Imaging, Canada) or Artemis (Wellcome Sanger Institute, UK) software from March 2020 to July 2023 at Yale New Haven Hospital and the affiliated Veterans Affairs Connecticut Healthcare System. Biopsies were performed transrectally by five urologists, and all patients received a periprostatic nerve block (PPNB). Pain scores were obtained by asking patients to rate their pain on a scale of 1–10, and were then recorded on the biopsy sheet by the biopsy nurse. Scores were recorded at specific points during the biopsy: viscous lidocaine (lido) instillation, PPNB, biopsy, and overall. Patients who did not have all four pain scores recorded were excluded, yielding a total of 779 individuals. The primary goal of this study was to assess the relationship between pain during lido instillation and PPNB with the overall pain experienced by patients undergoing prostate biopsy.

### 2.2. Periprostatic nerve block

Patients were positioned in the left lateral decubitus position to optimize procedural access. Local anesthesia was achieved with 10 mL of 2% lidocaine gel instilled into the rectum using an introducer. The PPNB was performed with an ultrasound probe aligned in the sagittal plane, and a 22-gauge, 7-inch spinal needle containing 5 mL of lidocaine was carefully inserted through the biopsy guide channel under ultrasound guidance. No sedation or additional analgesia was administered aside from the lidocaine gel and the standard PPNB (10 mL of lidocaine).

### 2.3. Statistical analysis

Data analysis was performed using Statistical Package for the Social Sciences software version 29 (IBM, United States). Clinical characteristics included age, body mass index (BMI), race, abnormal digital rectal examination (DRE) findings (defined as any prostate irregularity on physical examination), PSA, MRI-derived prostate volume, biopsy status, fusion score (defined as the highest Gleason Score identified between targeted and systematic biopsy), procedure time, history of anxiety, and history of chronic pain (obtained from the patients’ electronic medical records). Univariate analysis was conducted using non-parametric tests, including the Mann–Whitney U and Kruskal–Wallis H tests, to compare differences in categorical variables and overall pain levels. Pearson’s correlation coefficient was used to analyze the relationship between age and overall pain, whereas Spearman’s test was employed to evaluate correlations between continuous variables and overall pain. Multivariable logistic regression was performed to identify potential predictors of increased overall pain. Contemporary biopsy approaches, including targeted and systematic sampling, were not evaluated.

## 3. Results

A total of 779 eligible patients underwent fusion prostate biopsy, with a mean age of 67.2 years (range: 42.9–89.7) and a mean BMI of 28.17 kg/m^2^. Table 1 presents the patient demographics. The mean pain scores during lido instillation, PPNB, biopsy, and overall were 0.106, 2.79, 3.48, and 3.57, respectively. The mean biopsy duration lasted for 13 min (range: 5–59). Mean PSA levels were 9.9 ng/mL (range: 0.0120–192), with a median of 7.07 ng/mL. The median MRI-derived prostate volume was 55.10 mL (standard deviation: 38.1). Approximately 70.08% of the fusion biopsies were conducted using Artemis software (*n* = 546), whereas 29.9% were conducted using micro-ultrasound-guided biopsy (ExactVu) (*n* = 233). Approximately 570 patients had normal DRE findings, whereas 204 yielded abnormal DRE findings. In addition, 746 patients (95.76%) demonstrated benign prostatic hyperplasia on MRI. Regarding prior biopsy status, 452 patients (58.02%) underwent a prostate biopsy for the 1^st^ time (biopsy-naïve), 79 patients (10.14%) had a history of a prior negative biopsy, and 229 patients (29.39%) had previous positive biopsies and were undergoing AS.

**Table 1:** Results of univariate analysis

Variable	Mean	*n* (%)	Spearman’s correlation (*rho*)	Pearson’s correlation coefficient (*r*)	*p*-value	95% confidence interval	

						Lower	Upper
Clinical characteristics							
Age (SD)	67.2 (7.5)	-	-	−0.135	<0.001	−0.204	−0.065
BMI (SD)	28.17 (4.6)	-	−0.131	-	0.047	−0.259	0.002
PSA (median)	9.90 (7.07)	-	0.024	-	0.502	−0.048	0.096
MRI-derived prostate volume (median)	64.11 (55.10)	-	−0.002	-	0.967	−0.074	0.071
Total procedure duration	13 min	-	−0.136	-	<0.001	−0.196	−0.048
Race							
White	-	596 (76.5)	-	-	0.11	-	-
Black	-	83 (10.7)	-	-		-	-
Hispanic	-	24 (3.1)	-	-		-	-
Asian	-	16 (2.1)	-	-	-	-
Biopsy status							
Biopsy-naïve	-	452 (58)	-	-	0.002^a^	-	-
Prior negative	-	79 (10.1)	-	-		-	-
Active surveillance	-	229 (29.4)	-	-	-	-
Fusion score							
0	-	190 (24.4)	-	-	0.525	-	-
1	-	187 (24)	-	-		-	-
2	-	204 (26.2)	-	-		-	-
3	-	89 (11.4)	-	-		-	-
4	-	59 (7.6)	-	-		-	-
5	-	47 (6)	-	-	-	-
History of anxiety							
Yes	-	183 (23.5)	-	-	0.625	-	-
No	-	596 (76.5)	-	-	-	-
History of chronic pain							
Yes	-	195 (25)	-	-	0.249	-	-
No	-	584 (75)	-	-	-	-
Digital rectal examination							
Abnormal	-	204 (26)	-	-	0.002^a^	-	-
Normal	-	570 (73)	-	-		-	-
Software used							
Artemis	-	546 (70.08)	-	-	0.099	-	-
ExactVu	-	233 (29.9)	-	-	-	-
History of benign prostatic hyperplasia							
Yes	-	32 (4)	-	-	0.268	-	-
No	-	746 (96)	-	-		-	-

Note:

a represents a *p<*0.05. Abbreviations: BMI: Body mass index; MRI: Magnetic resonance imaging; PSA: Prostate-specific antigen; SD: Standard deviation.

### 3.1. Overall pain score

Univariate analysis revealed no significant difference in the mean overall pain score between patients who underwent biopsy using ExactVu software (3/10) and those who received biopsy using Artemis (4/10) (*p*=0.099). The mean overall pain scores differed by race (3 ± 1.9 for white vs. 4 ± 2.6 for black patients; *p*=0.006). Notably, patients on AS and patients with normal DRE findings demonstrated higher pain scores (4/10) compared with those with a prior negative biopsy, those who were biopsy-naïve, and those with abnormal DRE findings (3/10) (*p*=0.002 and *p*<0.001, respectively). In addition, older age (*r* = −0.135; *p*<0.001), BMI (*r_s_* = −0.131; *p*=0.047), and procedure duration showed significant, although weak, correlations with the overall pain score (*r_s_* = −0.136; *p*<0.001). These findings suggest that, as the procedure duration increases, the overall pain experienced by patients tends to slightly decrease.

In this study, 23.5% (183/779) of patients had a history of anxiety, and 25% (195/779) had a history of chronic pain. The Kruskal–Wallis test was employed to evaluate the relationship between these conditions and pain scores during fusion prostate biopsy. The findings revealed no significant differences in pain scores between patients with and without a history of anxiety during lido instillation (0.142 vs. 0.096; *p*=0.654), PPNB (2.99 vs. 2.73; *p*=0.186), biopsy (3.54 vs. 3.47; *p*=0.680), or overall (3.64 vs. 3.55; *p*=0.625). Similarly, patients with a history of chronic pain did not show significant differences in pain scores during lido instillation (0.154 vs. 0.091; *p*=0.559), PPNB (2.92 vs. 2.75; *p*=0.374), biopsy (3.73 vs. 3.40; *p*=0.091), or overall (3.77 vs. 3.51; *p*=0.249).

**Figure 1:**
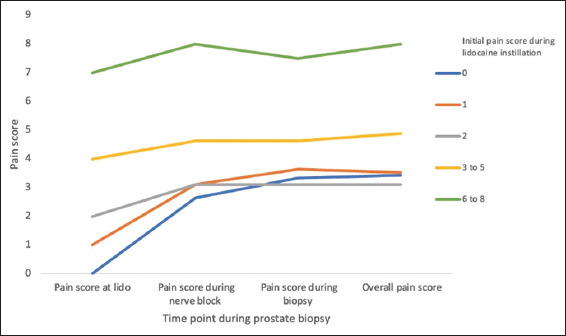
Pain scores (0–10) at different stages of the procedure, grouped by initial pain score during lido instillation. Abbreviation: Lido: Lidocaine.

Multivariable analysis (Table 2) revealed that black patients, compared with all other races (odds ratio [OR]: 2.838; *p*<0.001), and patients on AS (OR: 1.648; *p*=0.003), compared with biopsy-naïve patients, were more likely to experience the upper quartile (UQ) of overall pain. Men with abnormal DRE findings were less likely to suffer from the UQ of overall pain than their counterparts with normal DRE findings (OR: 0.586; *p*=0.004). A pain score during lido instillation of >2 (OR: 10.28; *p*=0.027) and a pain score during PPNB of >2 (OR: 7.49; *p*<0.001) increased the odds of reaching the UQ of overall pain (Figure 1). The cutoff of >2 was selected based on the sample distribution, as the 75^th^ percentile (UQ) of reported pain exceeded 2. Age (*p*=0.980), PSA (*p*=0.693), MRI-derived prostate volume (*p*=0.371), and fusion score (*p*=0.329) were not significantly correlated with overall pain.

**Table 2:** Binary logistic regression analysis of overall pain score

Variable	Odds ratio	95% confidence interval		*p*-value

		Lower	Upper	
ExactVu versus Artemis	1.33	0.952	1.858	0.095
Age	1.0	0.986	1.015	0.980
Race	-	-	-	<0.001
White^a^	-	-	-	-
Black	2.838^b^	1.812	4.629	<0.001^b^
Hispanic	1.678	0.715	3.941	0.234
Asian	2.605	0.918	7.394	0.072
Digital rectal examination	-	-	-	0.004
Abnormal^a^	-	-	-	-
Normal	0.586^b^	0.407	0.843	0.004^b^
MRI-derived prostate volume	0.998	0.994	1.002	0.371
PSA	1.003	0.989	1.016	0.693
Fusion score by grade group	-	-	-	0.329
1^a^	-	-	-	-
2	1.254	0.784	2.005	0.345
3	0.691	0.394	1.212	0.197
4	0.846	0.438	1.636	0.62
5	1.233	0.565	2.688	0.599
History of benign prostatic hyperplasia	0.833	0.341	2.033	0.688
History of anxiety	1.019	0.7	1.484	0.921
History of chronic pain	1.204	0.836	1.734	0.319
Biopsy status	-	-	-	0.035
Biopsy naïve^a^	-	-	-	-
Prior negative	1.088	0.632	1.872	0.761
Active surveillance	1.648^b^	1.126	2.379	0.003^b^

Notes:

a Reference point.

b represents a significant odds ratio determined by *p<*0.05. Abbreviations: MRI: Magnetic resonance imaging; PSA: Prostate-specific antigen.

## 4. Discussion

Previous studies have highlighted a broad spectrum of patient-related factors associated with discomfort and pain during prostate biopsy.^11^ Mitigation strategies that have been investigated include pre- and post-procedural analgesia, nerve blocks, topical anesthetic creams, sedation, nitrous oxide, diaphragmatic breathing, music therapy, and hand holding. Nevertheless, pain continues to be reported as an adverse effect of prostate biopsy and one that may decrease patient compliance over time.^10^,^12^ Factors such as the region of prostate biopsied, prostate anatomic dimensions, type of biopsy, and patient positions have previously been reported to contribute to increased pain during biopsy.^6^

In this study, patients on AS showed a significant association with higher pain levels compared with biopsy-naïve patients. This finding contrasts with previous studies, which suggest that patients undergoing their first biopsy may experience increased anxiety, heightened perceptions of pain, and greater anticipation of discomfort, likely due to concerns about oncological outcomes.^13^ In a prospective study of 319 patients, Sonmez *et al*.^11^ assessed the risk factors associated with pain during prostate fusion biopsy and identified a significant relationship between Visual Analog Scale scores and biopsy history, total prostate volume, and anorectal angle. Their findings indicated that biopsy-naïve patients, patients with a larger prostate, patients with a shorter prostate-anus surface distance, and those with a narrow anorectal angle were more likely to experience severe pain. In contrast, our cohort did not demonstrate a linear relationship between total prostate volume and pain levels, as MRI-derived prostate volume was not significantly associated with pain. However, variations in prostate volume measurements across imaging modalities can be considerable, potentially introducing additional statistical errors.^14^

In a retrospective study by Cebeci and Ozkan^15^ 477 patients were evaluated to assess clinical parameters for predicting pain. They found that abnormal rectal examination findings, the collection of more than 12 core samples, and the type of anesthesia used significantly predicted higher pain. Their findings contradict those of the present study, in which abnormal DRE findings were associated with less pain during prostate biopsy. This discrepancy may be ascribed to psychological factors, anesthetic effects, or differential pain responses.^16^

Psychological distress, particularly anxiety, has been identified in the literature as an important factor influencing pain perception and the patient experience during prostate biopsy.^17^ A prospective study by Krausewitz *et al.*,^17^ involving 108 patients demonstrated a significant correlation between pain and psychological factors, including anxiety, stress, and pain expectancy. In our cohort, however, no significant association was observed between anxiety and reported pain levels.

Patients undergoing biopsy tended to anticipate more pain than they ultimately experienced. To address these emotions, a non-randomized quality improvement project conducted by Grinberg *et al*.^18^ evaluated diaphragmatic breathing as an intervention during prostate biopsy and found a reduction in anxiety levels post-procedure. Implementing cost-effective, self-management strategies, such as diaphragmatic breathing, to improve the tolerability of transrectal ultrasound-guided prostate biopsy could represent an important approach to enhancing the overall patient experience. Other non-pharmacological approaches that have shown promise in pain reduction include mindfulness-based cognitive therapy, guided imagery, hand holding, and music therapy; however, further research is warranted to strengthen the evidence supporting these interventions. This underscores the potential of complementary, non-pharmacological strategies in pain management, which may improve patient comfort while reducing reliance on medications.^19^

The main objective of this study was to determine whether factors known before biopsy were associated with the pain experienced during the procedure. As rectal manipulation is similar between lido instillation and DRE, pain during lido instillation could serve as a surrogate for pain experienced at DRE. In addition, our findings indicated that a shorter waiting time following lido instillation was associated with higher initial pain scores. Understanding this relationship may help clinicians make informed decisions regarding preventative measures, such as scheduling a biopsy under sedation, thereby enhancing tolerability and improving the overall patient experience.

Nevertheless, several limitations remain in this study. The retrospective design limits control over how the original data were collected. Patient pain scores were primarily obtained through nurse documentation without a standardized tool, such as the Visual Analog Scale, leading to possible inconsistencies in timing, content, and measurement reliability throughout the procedure. To address this limitation, we limited our analysis to patients with complete documentation of all four pain scores, thereby improving consistency and comparability across the study population. Another limitation was the inability to control for additional confounders, such as the number of biopsy cores taken and variability in pain scores across the five urologists performing the procedure. The number of biopsy cores taken was determined by MRI findings, with 3–5 biopsy cores obtained for each target lesion. Moreover, this study included a low representation of Black patients, which limits the generalizability of the findings related to racial disparities; subgroup analyses or validation in larger, multicenter cohorts are warranted.

Future studies should continue to evaluate strategies that reduce pain, such as extending the time between administration of the PPNB and the start of the biopsy. For patients with risk factors for pain, it may be advisable to use more potent forms of anesthesia when feasible. Future research should also focus on designing a clinical decision-making tool that links DRE pain scores to sedation strategies, accompanied by a cost-effectiveness analysis to guide implementation. In addition, more robust counseling regarding biopsy expectations may help patients manage their anticipated discomfort. Integrating patient self-reported forms is recommended for a less biased recollection of pain data, and evaluating anxiety subtypes for formal analysis should be considered. Future studies should aim to conduct prospective trials incorporating these recommendations to provide more objective insights.

## 5. Conclusion

Among the patients who underwent fusion prostate biopsy, normal DRE findings, black patients, and ongoing AS were associated with higher overall pain scores. Notably, an initial pain score at lido instillation of more than two increased the odds of a UQ overall pain score. As this rectal manipulation is similar to the discomfort of a DRE, pain during DRE may serve as a useful indicator to better identify patients at the initial clinic visit who would benefit from sedation during prostate biopsy. This underscores the significance of early pain indicators in shaping the pain experience throughout the procedure. Further prospective, randomized trials are warranted to validate these findings.

## Data Availability

Data are available from the corresponding author upon reasonable request.
